# B7H3 ameliorates LPS-induced acute lung injury via attenuation of neutrophil migration and infiltration

**DOI:** 10.1038/srep31284

**Published:** 2016-08-12

**Authors:** Yan Li, Jie Huang, Niamh M. Foley, Yunyun Xu, Yi Ping Li, Jian Pan, H. Paul Redmond, Jiang Huai Wang, Jian Wang

**Affiliations:** 1 Institute of Paediatric Research, Affiliated Children’s Hospital, Soochow University, Suzhou, China; 2 Department of Paediatric Cardiology, Affiliated Children’s Hospital, Soochow University, Suzhou, China; 3 Department of Academic Surgery, University College Cork, Cork University Hospital, Cork, Ireland; 4 Department of Paediatric Surgery, Affiliated Children’s Hospital, Soochow University, Suzhou, China

## Abstract

Acute lung injury (ALI) and acute respiratory distress syndrome (ARDS) are characterized by an excessive inflammatory response within the lungs and severely impaired gas exchange resulting from alveolar-capillary barrier disruption and pulmonary edema. The costimulatory protein B7H3 functions as both a costimulator and coinhibitor to regulate the adaptive and innate immune response, thus participating in the development of microbial sepsis and pneumococcal meningitis. However, it is unclear whether B7H3 exerts a beneficial or detrimental role during ALI. In the present study we examined the impact of B7H3 on pulmonary inflammatory response, polymorphonuclear neutrophil (PMN) influx, and lung tissue damage in a murine model of lipopolysaccharide (LPS)-induced direct ALI. Treatment with B7H3 protected mice against LPS-induced ALI, with significantly attenuated pulmonary PMN infiltration, decreased lung myeloperoxidase (MPO) activity, reduced bronchoalveolar lavage fluid (BALF) protein content, and ameliorated lung pathological changes. In addition, B7H3 significantly diminished LPS-stimulated PMN chemoattractant CXCL2 production by inhibiting NF-κB p65 phosphorylation, and substantially attenuated LPS-induced PMN chemotaxis and transendothelial migration by down-regulating CXCR2 and Mac-1 expression. These results demonstrate that B7H3 substantially ameliorates LPS-induced ALI and this protection afforded by B7H3 is predominantly associated with its inhibitory effect on pulmonary PMN migration and infiltration.

Acute lung injury (ALI) and its more severe condition acute respiratory distress syndrome (ARDS) were first described in 1967^1^ and are characterized by an excessive inflammatory response within the lungs and severely impaired gas exchange resulting from alveolar-capillary barrier disruption and pulmonary edema^2^,^3^,^4^. Clinically, patients with ALI/ARDS present with acute onset, progressive hypoxemia, bilateral lung infiltrates on chest radiographs, increased work of breathing, and noncardiogenic respiratory failure^2^,^3^,^4^. ALI/ARDS is a common clinical disorder and presents substantial health problems worldwide. It has been estimated that ALI/ARDS affects one in 10 general intensive care unit patients, with 200,000 cases annually in the United States^2^,^3^,^4^,^5^. Although improved survival rates of ALI/ARDS have been observed during the past two decades, largely owing to advances in supportive critical care medicine^6^,^7^, mortality rates of ALI/ARDS remain high, ranging from 25% to 45% depending on various factors such as comorbidities^2^,^3^,^4^,^5^. Currently, there are no pharmacological approaches available for the treatment of ALI/ARDS, which highlights the urgent need in the development of therapeutic strategies for ALI/ARDS.

The B7 superfamily of costimulatory proteins plays an important role in the regulation of Ag-specific T cell-mediated immune responses^8^,^9^. B7H3, a newly discovered member of the B7 superfamily, has been identified in both humans and mice by sharing ∼88% amino acid sequence identity^10^,^11^. Accumulated evidence supports the notion that B7H3 possesses a contrasting role in regulating T cell-mediated immune responses by functioning as both a T cell costimulator and coinhibitor^11^,^12^,^13^,^14^. More recently, it has been shown that B7H3 is also involved in the innate immunity-associated inflammatory response. Although B7H3 is not expressed in significant amounts on freshly isolated human lymphocytes, it is induced in human monocytes/macrophages and dendritic cells upon inflammatory cytokine stimulation^11^,^12^,^13^,^15^. A soluble form of B7H3 (sB7H3), released from monocytes, dendritic cells, and activated T cells, is detectable in the circulation of healthy humans^16^. Our recent clinical studies found that patients diagnosed with sepsis^17^ and bacterial meningitis^18^ displayed significantly elevated levels of plasma and cerebrospinal fluid (CSF) sB7H3, and moreover, levels of sB7H3 in the circulation and CSF correlated closely with the disease status and clinical outcome in these patients^17^,^18^. In experimental LPS-induced septic shock^17^ and pneumococcal meningitis^19^, we further demonstrated that B7H3 augmented the TLR4 agonist lipopolysaccharide (LPS)- and gram-positive *Streptococcus pneumoniae* (*S. pneumoniae*)-stimulated proinflammatory cytokine production via both a TLR4- and TLR2-dependent manner. These results suggest that B7H3 functions as a costimulator of the innate immunity by augmenting the inflammatory response, and thus, may contribute to the development of microbial sepsis and pneumococcal meningitis.

In this study, we examined the influence of B7H3 on pulmonary inflammatory response, leukocyte influx, and lung edema in a murine model of LPS inhalation-induced direct ALI. Unexpectedly, we found that administration of B7H3 markedly attenuated LPS-induced lung injury, with significant reductions in pulmonary polymorphonuclear neutrophil (PMN) infiltration, lung myeloperoxidase (MPO) activity, and bronchoalveolar lavage fluid (BALF) protein content. In addition, treatment with B7H3 substantially inhibited LPS-stimulated overexpression and release of chemokine CXCL2 in the lungs. Furthermore, B7H3 *in vitro* significantly diminished LPS-stimulated murine alveolar macrophage (MAMϕ) CXCL2 production by inhibiting nuclear factor-kappaB (NF-κB) p65 phosphorylation and strongly attenuated LPS-induced PMN chemotaxis and transendothelial migration by down-regulating CXCR2 and Mac-1 expression.

## Results

### B7H3 attenuates PMN infiltration in the lungs and ameliorates lung tissue damage during LPS-induced ALI

**Figure 1:**
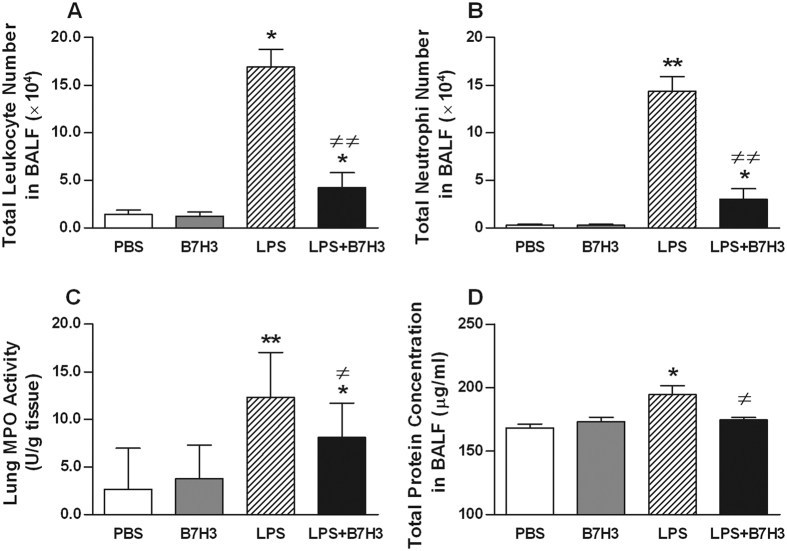
B7H3 attenuates LPS-induced PMN infiltration and lung tissue damage. Male Balb/c mice were randomized into PBS, B7H3, LPS, and LPS+B7H3 groups (n = 12 per group). BALF leukocyte (**A**) and PMN (**B**) counts, lung MPO activity (**C**), and BALF protein concentrations (**D**) were assessed 24 hrs after LPS inhalation as described in the Methods. Intranasal instillation of LPS (20 μg/mouse) resulted in significantly increased BALF leukocyte numbers, PMN counts, and protein concentrations as well as lung MPO activity, which were markedly attenuated by B7H3 treatment. Data are mean ± SD. *p < 0.05, **p < 0.01 versus PBS group; ^≠^*p* < 0.05, ^≠≠^*p* < 0.01 versus LPS group.

**Figure 2:**
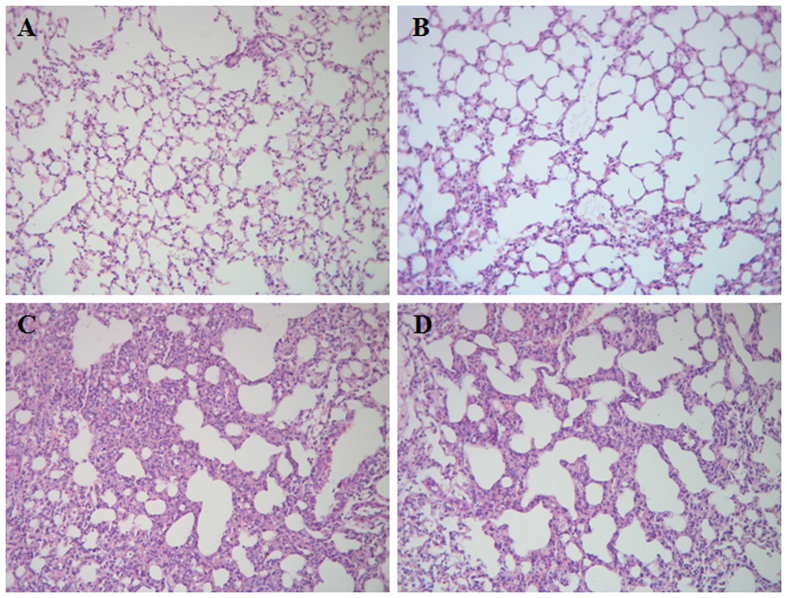
B7H3 ameliorates LPS-induced lung pathological alterations. Male Balb/c mice were randomized into PBS, B7H3, LPS, and LPS+B7H3 groups (n = 12 per group). Lung histological changes were assessed 24 hrs after LPS inhalation as described in the Methods. Representative images of haematoxylin and eosin stained sections of lung tissues for PBS (**A**), B7H3 (**B**), LPS (**C**), and LPS+B7H3 (**D**) are shown, indicating that treatment with B7H3 strongly ameliorates LPS-induced lung edema, haemorrhage, alveolar collapse, and PMN infiltration.

Significantly enhanced influx of leukocytes (*p* < 0.05) (Fig. 1A) and PMNs (*p* < 0.01) (Fig. 1B) into the lungs was observed in mice at 24 hrs after receiving an intranasal instillation of LPS compared with mice received an intranasal instillation of phosphate-buffered saline (PBS). MPO activity in lung tissues was also significantly increased in LPS-treated mice (*p* < 0.01 versus PBS-treated mice) (Fig. 1C). Administration of B7H3 at 2 hrs after LPS inhalation substantially attenuated LPS-induced leukocyte and PMN infiltration in the lungs by 75% and 79%, respectively, as well as lung MPO activity (*p* < 0.05, *p* < 0.01 versus LPS-treated mice) (Fig. 1A–C). Furthermore, LPS inhalation resulted in lung edema as evidenced by significantly elevated protein levels in BALF (*p* < 0.05 versus PBS-treated mice), which was markedly attenuated by B7H3 treatment (*p* < 0.05 versus LPS-treated mice) (Fig. 1D). Notably, B7H3 itself affected neither PMN influx nor BALF protein levels (Fig. 1). Histological examination of the lung tissues showed substantial morphological alterations including edema, haemorrhage, alveolar collapse, and PMN infiltration in LPS-treated mice, whereas treatment with B7H3 markedly ameliorated these pathological changes (Fig. 2).

### B7H3 down-regulates CXCL2, but not TNF-α, IL-1β, and IL-6, expression and release during LPS-induced ALI

**Figure 3:**
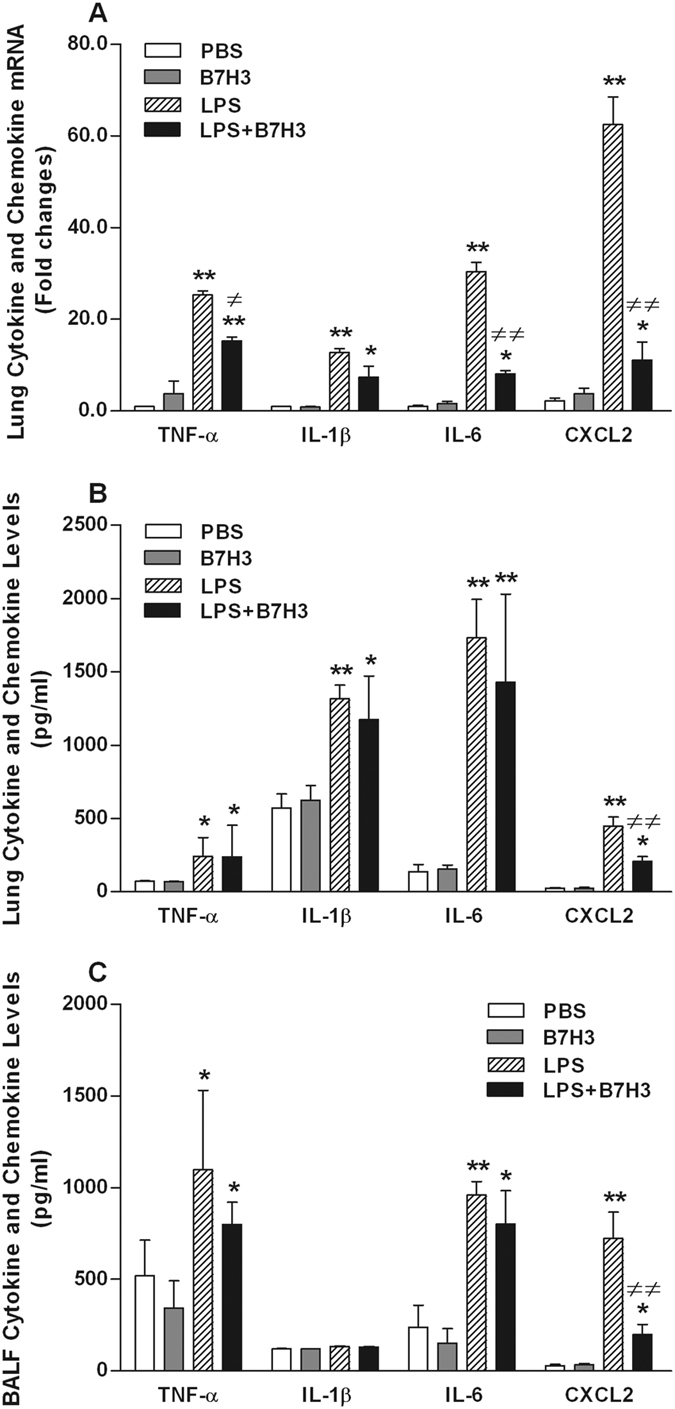
B7-H3 down-regulates LPS-stimulated CXCL2, but not TNF-α, IL-1β, and IL-6, expression and release. Male Balb/c mice were randomized into PBS, B7H3, LPS, and LPS+B7H3 groups (n = 12 per group). Lung mRNA (**A**) and protein (**B**) expression and BALF levels (**C**) of TNF-α, IL-1β, IL-6, and CXCL2 were measured 24 hrs after LPS inhalation as described in the Methods. Remarkably, treatment with B7H3 down-regulated LPS-stimulated chemokine CXCL2 expression in the lung and attenuated its release into the BALF. Data are mean ± SD. *p < 0.05, **p < 0.01 versus PBS group; ^≠^*p* < 0.05, ^≠≠^*p* < 0.01 versus LPS group.

Following LPS inhalation, there were significant increases in proinflammatory cytokines TNF-α, IL-1β, IL-6 and chemokine CXCL2 at both mRNA (Fig. 3A) and protein (Fig. 3B) levels in the lungs (*p* < 0.05, *p* < 0.01 versus PBS-treated mice). Moreover, LPS inhalation resulted in substantially increases of TNF-α, IL-6, and CXCL2 levels in BALF (*p* < 0.05, *p* < 0.01 versus PBS-treated mice) (Fig. 3C). Treatment with B7H3 markedly attenuated LPS-stimulated CXCL2 expression in the lungs and release into the BALF (*p* < 0.01 versus LPS-treated mice) (Fig. 3). Although B7H3 significantly attenuated LPS-stimulated TNF-α and IL-6 mRNA expression (*p* < 0.05, *p* < 0.01 versus LPS-treated mice) (Fig. 3A), it had no inhibitory effects on LPS-stimulated TNF-α and IL-6 protein expression and release (Fig. 3B,**C**).

### B7H3 does not affect PMN apoptosis, but diminishes LPS-stimulated PMN ROS generation

**Figure 4:**
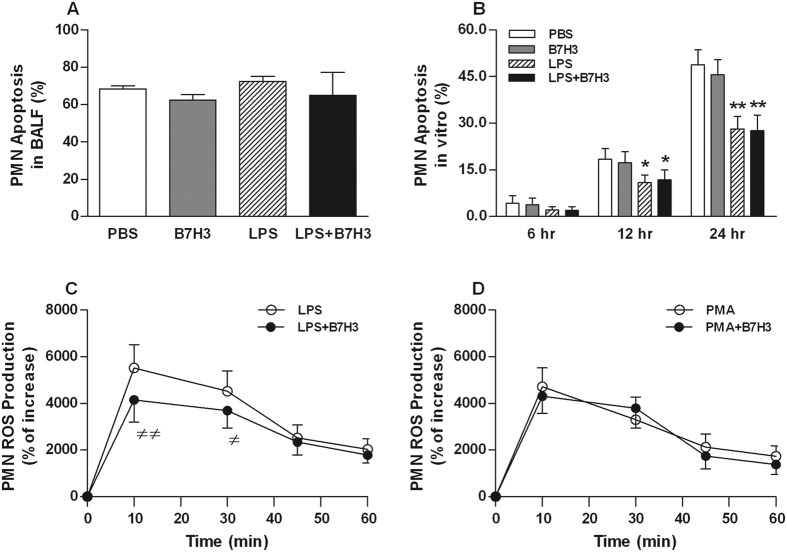
The effect of B7-H3 on PMN apoptosis and ROS production. (**A,B**) PMN apoptosis in BALF collected 24 hrs after LPS inhalation (n = 12 per group) or PMN apoptosis *in vitro* at indicated time points after PMNs treated with PBS, B7H3, LPS, and LPS+B7H3 (n = 8 per group) were assessed as described in the Methods. Data are mean ± SD. (**C,D**) ROS production was determined after PMNs treated with LPS and LPS+B7H3 or PMA and PMA+B7H3 as described in the Methods. B7H3 had no effect on PMN apoptosis both *in vitro* and *in vivo*, but significantly diminished LPS-induced ROS generation in PMNs. Data are mean ± SD of seven to eight independent experiments and each experiment was conducted in duplicate. *p < 0.05, **p < 0.01 versus PBS group; ^≠^*p* < 0.05, ^≠≠^*p* < 0.01 versus LPS group.

There were no statistically significant differences found in apoptotic rates of PMNs collected from BALF among PBS-treated, B7H3-treated, LPS-treated, and LPS + B7H3-treated mice (Fig. 4A). Moreover, although *in vitro* LPS stimulation led to a significant delay in PMN spontaneous apoptosis after incubation of PMNs with LPS for 12 and 24 hrs (*p* < 0.05, *p* < 0.01 versus PMNs incubated with PBS), the addition of B7H3 did not show any modulatory effects on LPS-delayed PMN spontaneous apoptosis (Fig. 4B).

Next, we examined the influence of B7H3 on PMN reactive oxygen species (ROS) production, as represented by superoxide anion generation in LPS-stimulated PMNs. As shown in Fig. 4C, stimulation of PMNs with LPS resulted in a substantial increase in superoxide anion generation; however, the addition of B7H3 significantly attenuated LPS-stimulated ROS production (*p* < 0.05, *p* < 0.01 versus LPS-stimulated PMNs). In contrast, B7H3 had no inhibitory effects on PMA-stimulated PMN superoxide anion generation (Fig. 4D).

### B7H3 attenuates LPS-induced PMN chemotaxis and transendothelial migration via down-regulation of CXCR2 and Mac-1 expression

**Figure 5:**
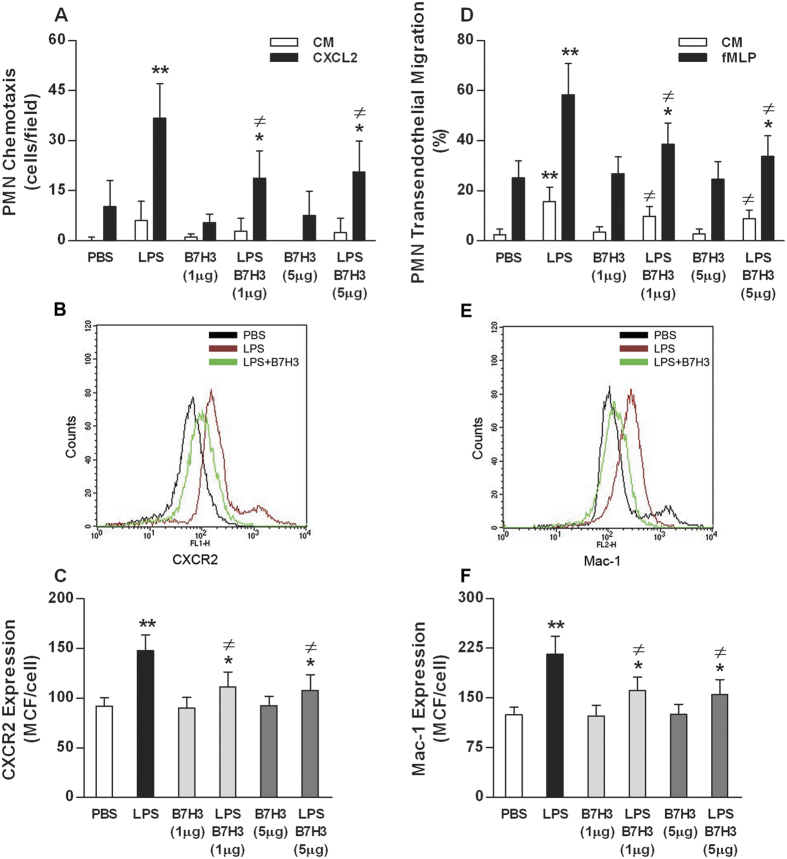
B7H3 attenuates LPS-induced PMN chemotaxis and transendothelial migration by down-regulating CXCR2 and Mac-1 expression. PMN chemotaxis toward CXCL2 (**A**) and migration across the MPEC monolayer (**D**) were assessed as described in the Methods. Surface expression of CXCR2 (**B,C**) and Mac-1 (**E,F**) on PMNs was detected by FACScan analysis. The addition of B7H3 significantly attenuated LPS-induced PMN chemotaxis and transendothelial migration toward the chemoattractants CXCL2 and fMLP, which are closely associated with the inhibitory effect of B7H3 on LPS-induced up-regulation of CXCR2 and Mac-1 expression. Data are mean ± SD of six to eight independent experiments and each experiment was conducted in duplicate. *p < 0.05, **p < 0.01 versus PBS group; ^≠^*p* < 0.05 versus LPS group.

We first examined the ability of PMN chemotaxis toward the chemoattractant CXCL2. LPS stimulation resulted in significantly enhanced PMN chemotaxis (*p* < 0.01 versus PBS-treated PMNs) (Fig. 5A), which correlated with LPS-induced up-regulation of CXCR2 expression on PMNs (*p* < 0.01 versus PBS-treated PMNs) (Fig. 5B,**C**). However, the LPS-induced increase in PMN chemotaxis was strongly attenuated by B7H3 at both 1.0 and 5.0 μg/ml concentrations (*p* < 0.05 versus LPS-stimulated PMNs) (Fig. 5A), and moreover, the LPS-induced up-regulation of PMN CXCR2 expression was also substantially inhibited by B7H3 (*p* < 0.05 versus LPS-stimulated PMNs) (Fig. 5B,**C**).

We further assessed PMN migration across the murine pulmonary endothelial cell (MPEC) monolayer in response to the chemoattractant fMLP. As shown in Fig. 5D, stimulation of PMNs with LPS significantly enhanced their transendothelial migration (*p* < 0.01 versus PBS-treated PMNs), with more PMN migration toward the chemoattractant fMLP than toward culture medium. Moreover, LPS stimulation markedly up-regulated surface expression of Mac-1 on PMNs (*p* < 0.01 versus PBS-treated PMNs) (Fig. 5E,F). Notably, the addition of B7H3 substantially attenuated LPS-stimulated PMN transendothelial migration (*p* < 0.05 versus LPS-treated PMNs) (Fig. 5D) and Mac-1 expression (*p* < 0.05 versus LPS-treated PMNs) (Fig. 5E,F).

### B7H3 inhibits LPS-stimulated MAMϕ CXCL2 expression through attenuation of NF-κB p65 phosphorylation

**Figure 6:**
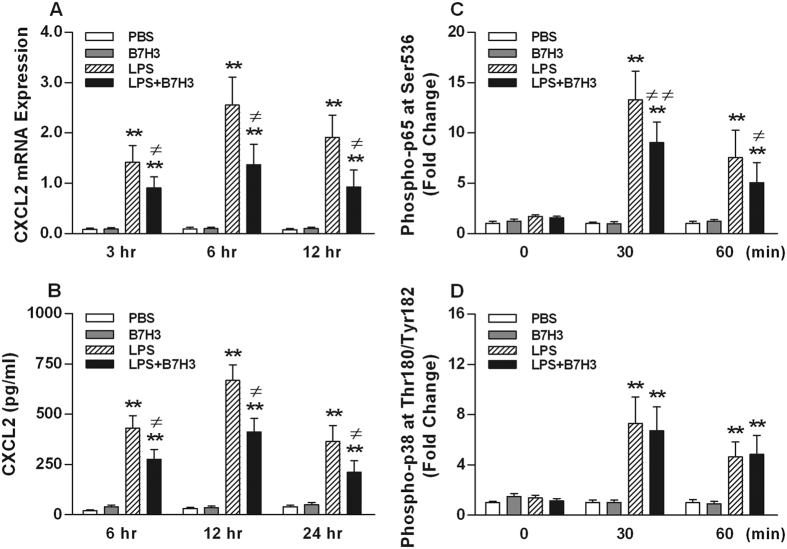
B7H3 inhibits LPS-stimulated MAMϕ CXCL2 expression by attenuating NF-κB p65 phosphorylation. MAMϕs were incubated with PBS, B7H3, LPS, and LPS+B7H3 for various time periods. CXCL2 mRNA (**A**) and protein (**B**) expression was assessed as described in the Methods. Intracellular expression of phophorylated NF-κB p65 (**C**) and MAPK p38 (**D**) was detected by FACScan analysis. B7H3 strongly down-regulated LPS-stimulated CXCL2 expression in MAMϕs at both mRNA and protein levels, and this correlates with the attenuation of LPS-induced phosphorylation of NF-κB p65, but not MAPK p38, by B7H3. Data are mean ± SD of six to eight independent experiments and each experiment was conducted in duplicate. **p < 0.01 versus PBS group; ^≠^*p* < 0.05, ^≠≠^*p* < 0.01 versus LPS group.

As shown in Fig. 6A,B, there were significant increases in CXCL2 expression at both mRNA and protein levels observed in LPS-stimulated MAMϕs at different time points after LPS stimulation (*p* < 0.01 versus PBS-treated MAMϕs). This up-regulated expression of CXCL2 correlated with LPS-induced phosphorylation of NF-κB p65 and MARK p38 (*p* < 0.01 versus PBS-treated MAMϕs) (Fig. 6C,D). Incubation of MAMϕs with B7H3 significantly attenuated LPS-stimulated CXCL2 mRNA expression (Fig. 6A) and protein production (Fig. 6B) (p < 0.05 versus LPS-treated MAMϕs), and inhibited LPS-induced activation of NF-κB p65, but not MARK p38 (*p* < 0.05 versus LPS-treated MAMϕs) (Fig. 6C,D). B7H3 itself did not affect the expression of CXCL2 and phosphorylation of NF-κB p65 and MARK p38 (Fig. 6).

## Discussion

B7H3, a newly discovered member of the B7 superfamily of costimulatory proteins, possesses a contrasting role in regulating the adaptive immune response by functioning as both a T cell costimulator and coinhibitor^11^,^12^,^13^,^14^. More recently, it has been shown that B7H3 also participates in the innate immune response by acting as a costimulator of innate immunity to augment inflammatory responses during experimental septic shock and pneumococcal meningitis^17^,^19^. In the current study, we investigated the impact of B7H3 on pulmonary inflammatory response, PMN influx into the lungs, and lung tissue damage in a murine model of LPS-induced direct ALI. We reported that B7H3 effectively protected mice against LPS-induced lung injury, with significantly attenuated pulmonary PMN infiltration, decreased lung MPO activity, reduced BALF protein content, and ameliorated lung pathological changes. We further demonstrated that B7H3 significantly inhibited LPS-stimulated overexpression and release of PMN chemoattractant CXCL2 through attenuation of NF-κB p65 activation, and substantially attenuated LPS-induced PMN chemotaxis and transendothelial migration via down-regulation of CXCR2 and Mac-1 expression.

Exuberant infiltration and accumulation of leukocytes, in particular PMNs in both interstitial and alveolar spaces of the lungs is one of the most important pathological hallmarks of ALI^2^,^20^,^21^. Although a quick and appropriate influx of PMNs from the circulation into the lungs is essential for clearance of microbial pathogens and debris from the alveolar space, excessive and persistent sequestration of PMNs may cause additional injury to the lungs by release of several toxic mediators including ROS, proteases, proinflammatory cytokines, and procoagulant molecules, thus exacerbating ALI^20^,^22^,^23^. It has been reported that the number of PMNs in the BALF of patients with ARDS correlates closely with the severity and poor outcome of disease^20^,^24^, while depletion of PMNs in animal models of LPS-induced ALI ameliorates lung injury with reduced endothelial-epithelial damage and capillary-alveolar permeability^25^,^26^. In the present study, an intranasal instillation of LPS in mice led to markedly enhanced PMN infiltration and MPO activity in the lungs, accompanied by lung edema and tissue damage. Treatment with B7H3 significantly attenuated LPS-induced influx of PMNs into the lungs, thus ameliorating lung edema and injury.

We next examined the underlying mechanism(s) by which B7H3 attenuates LPS-induced PMN infiltration and accumulation in the lungs. It has been shown that PMN recruitment from the circulation into the site of inflammation depends predominantly on the chemokine receptor CXCR2, and reduced CXCR2 expression on circulating PMNs is associated with diminished PMN influx into the infectious site^27^,^28^. We found that B7H3 treatment significantly inhibited LPS-stimulated up-regulation of CXCR2 expression on PMNs, thus attenuating LPS-induced PMN chemotaxis toward the chemoattractant CXCL2. We further demonstrated that B7H3 down-regulated LPS-induced overexpression of Mac-1, a critical adhesion molecule responsible for PMN extravasation via transendothelial migration^29^, and consequently, attenuated PMN transmigration across the MPEC monolayer. The chemoattractant CXCL2 plays a key role in PMN recruitment, and BALF obtained from patients with ARDS has been shown to be highly chemotactic for human PMNs^27^,^30^. We found that LPS inhalation resulted in markedly increased CXCL2 expression in the lungs and elevated CXCL2 concentrations in the BALF, whereas B7H3 treatment significantly reduced LPS-stimulated CXCL2 production and release into the BALF. Delayed spontaneous apoptosis of PMNs sequestrated in the lungs and subsequently released toxic mediators including ROS by these PMNs have been shown to contribute to the development and progression of ALI and/or ARDS^20^,^31^. We found that B7H3 treatment did not affect apoptotic rates of PMNs in the BALF or LPS-induced delay in PMN spontaneous apoptosis; however, it substantially reduced LPS-stimulated PMN ROS production.

Our previous work has revealed that B7H3, in addition to regulating T cell-mediated immune responses, acts as a costimulator of innate immunity by augmenting proinflammatory cytokine and chemokine production in monocytes/macrophages and microglial cells^17^,^19^. In this study, we further examined the influence of B7H3 on pulmonary inflammatory response in a murine model of LPS-induced direct ALI. LPS inhalation led to markedly increased expression and production of proinflammatory cytokines TNF-α, IL-1β, IL-6, and chemokine CXCL2 in the lungs. Surprisingly, B7H3 strongly attenuated LPS-stimulated CXCL2 expression at both mRNA and protein levels as well as its release into the BALF. We then analyzed the effect of B7H3 on NF-κB p65 and MARK p38 activation in LPS-stimulated MAMϕs. B7H3 substantially inhibited LPS-induced NF-κB p65 phosphorylation, thereby attenuating CXCL2 expression and production in LPS-stimulated MAMϕs. Notably, B7H3 also significantly attenuated LPS-stimulated TNF-α and IL-6 mRNA expression in the lungs, although it had no inhibitory effect on TNF-α and IL-6 protein expression.

It has been well documented that B7H3, being both a T cell costimulator and coinhibitor, plays a contrasting role in regulation of T cell-mediated immune responses^11^,^12^,^13^,^14^, indicating its involvement in autoimmunity and self tolerance^32^. In addition to the adaptive immunity, B7-H3 has been shown to promote the innate immunity-associated inflammatory response by augmenting proinflammatory cytokine and chemokine production in monocytes/macrophages and microglial cells^17^,^19^, thus participating in the development of experimental sepsis and pneumococcal meningitis. The accumulated evidence has revealed that elevated levels of B7H3 in certain human tumours correlate closely with poor clinical outcome including survival, prognosis, and tumour grade^33^,^34^; however, B7H3 also possesses strong antitumour effects^34^,^35^, suggesting the dual functions B7H3 has in tumour immunity. Interestingly, we demonstrated an inhibitory action of B7H3 on PMNs in the present study, which is largely in contrast to the previously observed stimulatory effect of B7H3 on monocytes/macrophages and microglial cells^17^,^19^. These somewhat discordant results indicate that B7H3 acts as not only a costimulator but also a coinhibitor during the innate immunity-initiated inflammatory response, which might be due to the existence of two receptors for B7H3 with opposing functions^32^, probably dependent on the targeted innate effector cells.

Taken together, in the current study we demonstrate that B7H3 strongly attenuates PMN infiltration and sequestration in the lungs, thus protecting mice against LPS-induced ALI. We have further revealed that B7H3 significantly diminishes LPS-stimulated PMN chemoattractant CXCL2 production by inhibiting NF-κB p65 phosphorylation and substantially reduces LPS-induced PMN chemotaxis and transendothelial migration by down-regulating CXCR2 and Mac-1 expression, which could be the two underlying mechanisms responsible for B7H3-attenuated pulmonary PMN infiltration and accumulation.

## Methods

### Regents and mAbs

LPS (*E. coli* serotype O55B5), fibronectin, PMA, fMLP, and recombinant mouse B7H3 were purchased from Sigma-Aldrich (St. Louis, MO, USA) and R&D Systems (Minneapolis, MN, USA), respectively. PE-conjugated anti-phospho p65 and anti-phospho p38 MAPK mAbs were obtained from Cell Signaling Technology (Beverly, MA, USA). PE- or FITC-conjugated anti-CXCR2 and anti-Mac-1 mAbs were purchased form R&D systems and BD Bioscience (San Jose, CA, USA), respectively. Endothelial cell basal medium-2 (EBM-2) MV and endothelial growth medium-2 (EGM-2) were obtained from PromoCell (Heidelberg, Germany), and other culture medium and reagents used for cell cultures were purchased from Invitrogen Life Technologies (Paisley, Scotland, U.K.). All other chemicals, unless indicated, were from Sigma-Aldrich.

### Mice and LPS-induced ALI

Pyrogen-free, 8- to 10-week old male Balb/c mice were purchased from Slac (Shanghai, China). Mice were housed in barrier cages under controlled environmental conditions (12/12 h of light/dark cycle, 55% ± 5% humidity, 23 °C) in the Institute of Paediatric Research, Soochow University and had free access to standard laboratory chow and water. All animal studies were approved by the institutional animal care and use committee at Soochow University and complied with the animal welfare act. The methods applied in this study were carried out in accordance with the approved guidelines.

Age- and weight-matched male Balb/c mice (n = 48) were anesthetized by intramuscular injection of ketamine/xylazine (80/10 mg/kg) admixture and randomized into one of the following 4 study groups: PBS, B7H3, LPS, and LPS + B7H3 (n = 12 per group). ALI was induced by intranasal instillation of LPS (20 μg/mouse in 100 μl PBS) as described previously^36^. Mice in PBS and B7H3 groups received intranasal instillation of PBS and B7H3 (5 μg/mouse), respectively, while mice in LPS + B7H3 group received B7H3 at 2 hrs after LPS inhalation. The experiment was terminated at 24 hrs after LPS inhalation, and lung tissues and BALF were harvested and collected thereafter. The *in vivo* study was carried out in two separate experiments.

### Measurement of leukocyte influx and protein content in BALF

BALF was collected after the lungs were lavaged with 1 ml PBS for three times as described previously^37^. Total and differential leukocyte counts in BALF were carried out using a standard haemocytometer and Wright-Giemsa staining of cytospin preparations, respectively. Total protein concentrations in BALF were determined using a BCA protein assay kit (Piece, Rockford, IL, USA).

### Assessment of MPO activity

MPO activity in the lungs was assessed as described previously^38^. Briefly, lung tissues were collected and homogenized in KPO_4_ buffer containing 0.5% hexa-decyl-trimethyl-ammonium bromide. After centrifugation, the resultant supernatant was diluted in reaction solution containing o-dianisidine hydrochloride and hydrogen peroxide. Changes in optical density (OD) were measured at 460 nm to calculate MPO activity.

### Lung histological examination

The harvested lung tissues were fixed in 4% paraformaldehyde, embedded in paraffin, cut into 4-μm sections, and stained with haematoxylin and eosin. Morphological changes in lung tissues were evaluated by light microscopy in a blinded fashion.

### Measurement of inflammatory cytokines and chemokine

TNF-α, IL-1β, IL-6, and CXCL2 mRNA expression in the lungs and MAMϕs were assessed by quantitative real-time RT-PCR as described previously^19^. Briefly, Total RNA was extracted from lung tissues and MAMϕs using a GeneElute^TM^ mammalian total RNA purification kit (Sigma-Aldrich) and reverse-transcribed into cDNA using the SuperScript first-strand synthesis system (Invitrogen). Amplification of cDNA was conducted using a LightCycler system (Roche Molecular Biochemicals, Indianapolis, IN, USA). The target gene mRNA expression was normalized with the housekeeping gene β-actin. The gene-specific primers used in this study were listed in Table 1. Concentrations of TNF-α, IL-1β, IL-6, and CXCL2 in BALF, lung tissues, and MAMϕ supernatants were quantitatively measured by ELISA (eBioscience, San Diego, CA, USA) according to the manufacturer’s instructions.

### Isolation and culture of primary murine PECs, AMϕ, and PMNs

MPECs were isolated using the microbead technique as described previously^39^. Briefly, harvested lungs from adult Balb/c mice were minced, digested, and filtered through a 100-μm nylon mesh. The resultant cell suspension was incubated with the anti-mouse CD31 Ab (BD Biosciences) and anti-rat IgG-coated micromagnetic beads (MACS Miltenyl Biotec Ltd., Surrey, U.K.). Cells captured were cultured with EBM-2 containing EGM-2 MV and 20% FCS in fibronectin-coated flasks at 37^o^C, 5% CO_2_ conditions. This procedure yielded the MPEC monolayer with >90% homogeneity. MPECs from first to third passage were used for experiments. MAMϕs were obtained from BALF as described previously^40^. Briefly, the lungs of adult Balb/c mice were lavaged three times with 1 ml PBS containing 2 mM EDTA. The collected BALF was centrifuged, and the resultant cell pellet was resuspended and incubated with DMEM containing 10% FCS in 24-well plates for 60 min to remove the non-adherent cells. This procedure yielded MAMϕs with >95% purity. PMNs were isolated from the bone marrow of adult Balb/c mice by discontinuous Percoll gradient centrifugation as described previously^41^.

### Assays for PMN apoptosis and ROS production

Apoptotic rates of PMNs collected from BALF and PMNs incubated with PBS, B7H3 (1.0 μg/ml), LPS (100 μg/ml), and LPS + B7H3 (100 + 1.0 μg/ml) for various time periods were assessed using an Annexin V apoptosis detection kit (eBioscence).

PMN ROS production was assessed with the fluorogenic substrate lucigenin (bis-*N*-methylacridinium nitrate) (Sigma-Aldrich) as described previously^42^. Briefly, murine PMNs were incubated with LPS (100 μg/ml) or LPS + B7H3 (100 + 1.0 μg/ml) for 60 min. PMNs were also incubated with PMA (1.0 μg/ml) to measure the maximal receptor-independent ROS production.

### Assessment of PMN chemotaxis and transendothelial migration

PMN chemotaxis was assessed as described previously^43^. Briefly, murine PMNs were incubated with PBS, B7H3 (1.0 and 5.0 μg/ml), LPS (100 μg/ml), and LPS + B7H3 (100 μg/ml + 1.0 and 5.0 μg/ml) for 60 min, plated onto 48-well chemotaxis plates (NeuroProbe, Gaithersburg, MD, USA), and allowed to migrate toward culture medium or CXCL2 (30 ng/ml) (R&D Systems) for 60 min.

PMN transendothelial migration was assessed as described previously^44^. Briefly, MPECs were grown to form the monolayer on fibronectin-coated Transwell inserts (3.0-μm-diameter pores) (BD Biosciences). Murine PMNs preincubated with PBS, B7H3, LPS, and LPS + B7H3 for 60 min were added into the upper chamber, and fMLP (10^−8^ M) was added into the lower chamber. After PMNs were allowed to migrate for 3 hrs, non-migrated PMNs (upper chamber) and migrated PMNs (lower chamber) were collected and counted.

### FACScan analysis for CXCR2 and Mac-1 expression

Murine PMNs were incubated with PBS, B7H3 (1.0 and 5.0 μg/ml), LPS (100 μg/ml), and LPS + B7H3 (100 μg/ml + 1.0 and 5.0 μg/ml) for 60 min, and stained with anti-CXCR2 and anti-Mac-1 mAbs conjugated with PE or FITC. PE- or FITC-conjugated isotype-matched mAbs were used as the control. FACScan analysis was performed from at least 10,000 events for detecting the surface expression of CXCR2 and Mac-1 on PMNs.

### Assays for NF-κB p65 and MAPK p38 phosphorylation

Primary MAMϕs were incubated with PBS, B7H3 (1.0 μg/ml), LPS (100 μg/ml), and LPS + B7H3 (100 + 1.0 μg/ml) for various time periods. Cells were fixed and permeabilised with Phosflow fix buffer and Phosflow perm buffer (BD Biosciences) for 30 min on ice. Cells were then stained with PE-conjugated anti-phospho p65 and anti-phospho p38 MAPK mAbs. PE-conjugated isotype-matched mAbs were used as the control. FACScan analysis was performed from at least 10,000 events for detecting the fold change of intracellular phosphorylated p65 or phosphorylated p38 expression.

### Statistical analysis

All data are presented as the mean ± SD. Statistical analysis was performed with GraphPad software version 5.01 (Prism, La Jolla, CA, USA). Comparison between groups was carried out using one-way ANOVA, followed by Bonferroni correction when necessary, and Mann-Whitney *U* test, where appropriate. Differences were judged to be statistically significant when the *p* value was less than 0.05.

## Additional Information

**How to cite this article**: Li, Y. *et al*. B7H3 ameliorates LPS-induced acute lung injury via attenuation of neutrophil migration and infiltration. *Sci. Rep.* **6**, 31284; doi: 10.1038/srep31284 (2016).

## Figures and Tables

**Table 1:** Gene-specific PCR primers.

	Product length (bp)	Sense	Antisense
Mouse TNF-α	122	CTGAACTTCGGGGTGATCGG	GGCTTGTCACTCGAATTTTGAGA
Mouse IL-1β	116	GAAATGCCACCTTTTGACAGTG	TGGATGCTCTCATCAGGACAG
Mouse IL-6	131	CTGCAAGAGACTTCCATCCAG	AGTGGTATAGACAGGTCTGTTGG
Mouse CXCL2	108	CCAACCACCAGGCTACAGG	GCGTCACACTCAAGCTCTG
β-actin	211	GTGACGTTGACATCCGTAAAGACC	ATCTGCTGGAAGGTGGACAGTGAG
